# The Tricyclodecan-9-yl-xanthogenate D609 Triggers Ceramide Increase and Enhances FasL-Induced Caspase-Dependent and -Independent Cell Death in T Lymphocytes

**DOI:** 10.3390/ijms13078834

**Published:** 2012-07-16

**Authors:** Delphine Milhas, Nathalie Andrieu-Abadie, Thierry Levade, Hervé Benoist, Bruno Ségui

**Affiliations:** 1 Team 4, Cancer Research Center of Toulouse, INSERM UMR1037, BP84225, 31432 Toulouse Cedex 4, France; E-Mails: delphinemilhas@yahoo.fr (D.M.); nathalie.andrieu@inserm.fr (N.A.-A.); thierry.levade@inserm.fr (T.L.); herve.benoist@inserm.fr (H.B.); 2 Department of Cell Biology, Hematology and Immunology, Faculty of Pharmaceutical Sciences, Paul Sabatier University (Toulouse III), 31062 Toulouse, France

**Keywords:** CD95, apoptosis, necrosis, sphingomyelin synthase, glucosylceramide synthase, ALPS

## Abstract

D609 is known to modulate death receptor-induced ceramide generation and cell death. We show that in Jurkat cells, non-toxic D609 concentrations inhibit sphingomyelin synthase and, to a lesser extent, glucosylceramide synthase, and transiently increase the intracellular ceramide level. D609 significantly enhanced FasL-induced caspase activation and apoptosis. D609 stimulated FasL-induced cell death in caspase-8-deficient Jurkat cells, indicating that D609 acts downstream of caspase-8. At high FasL concentration (500 ng/mL), cell death was significantly, but not completely, inhibited by zVAD-fmk, a broad-spectrum caspase inhibitor, indicating that FasL can activate both caspase-dependent and -independent cell death signaling pathways. FasL-induced caspase activation was abolished by zVAD-fmk, whereas ceramide production was only partially impaired. D609 enhanced caspase-independent ceramide increase and cell death in response to FasL. Also, D609 overcame zVAD-fmk-conferred resistance to a FasL concentration as low as 50 ng/mL and bypassed RIP deficiency. It is likely that mitochondrial events were involved, since Bcl-xL over-expression impaired D609 effects. In PHA-activated human T lymphocytes, D609 enhanced FasL-induced cell death in the presence or absence of zVAD-fmk. Altogether, our data strongly indicate that the inhibition of ceramide conversion to complex sphingolipids by D609 is accompanied by an enhancement of FasL-induced caspase-dependent and -independent cell death in T lymphocytes.

## 1. Introduction

D609 is a xanthate compound with anti-viral, anti-tumor and anti-inflammatory properties [1–5]. D609 has been widely used as an inhibitor of phosphatidylcholine-specific phospholipase C (PC-PLC) [2,6–9] and, indirectly, acidic sphingomyelinase (SMase) [2,4,10–14]. For instance, D609 impairs TNF- and anti-Fas-induced activation of a PC-PLC (the molecular identity of which remains unknown) and acidic SMase [4,10,11]. In this context, PC-PLC stimulation leads to the rise of diacylglycerol (DAG), which enhances acidic SMase activity and hence ceramide levels [10,12]. More recently, D609 has been shown to be being capable of inhibiting sphingomyelin synthase (SMS) [15–18], an enzyme that regulates ceramide and DAG levels in the opposite direction [17]. In addition, as a reducing agent, D609 could scavenge reactive oxygen species (ROS) [19].

D609 inhibits cell proliferation of different cell types, including cancer cells [1–5]. Whereas the molecular mechanisms involved in the anti-proliferative effects remain to be fully established, ceramide elevation as a consequence of SMS inhibition likely contribute to the up-regulation of cyclin-dependent kinase inhibitor p21 and cell cycle inhibition triggered by D609 [20]. However, one should note that inhibition of SMS by D609 has been recently reported to restore cell cycle progression in 2-hydroxyoleic acid-treated human glioma cells [21]. Thus, the effect of D609 on cell proliferation is likely to be cell type-dependent and/or may be dependent on the cell culture conditions.

D609 has been reported to modulate cell death signaling initiated by death receptors. D609 prevents TNF-induced cytotoxicity in various cell types both *in vitro* and *in vivo* [4,22]. However, D609 has been shown to sensitize U937 leukemia cells to TNF and an agonistic anti-Fas antibody [23]. Controversy exists as to the effect of D609 in Fas signaling. Okazaki’s group reported that D609 inhibits a nuclear SMS activity and enhances Fas cross-linking-induced ceramide production and cell death in Jurkat cells [16]. More recently, it has been published that D609 impaired HeLa cell death in response to an agonistic anti-Fas antibody, whereas it had no effect in SKW6.4 cells [24]. Thus, one can speculate that the ability of D609 to modulate death receptor-induced cell death is cell type-dependent. However, it should be noted that opposite findings were reported using the same cell type, *i.e.*, Jurkat cells, in response to Fas engagement [16,24].

Scaffidi and co-workers reported the existence of two different cell types as defined by distinct Fas signaling routes [25]. Type 1 cells were originally defined by their capacity to form large amounts of death-inducing signaling complex (DISC) consisting of the recruitment of the adaptor protein FADD and initiator caspases to the Fas receptor upon activation. This enables strong and direct caspase cascade activation, independent of mitochondrial events. In type 2 cells, DISC formation occurs less efficiently than in type 1 cells. Both initiator caspases, *i.e.*, caspase-8 and -10, are activated at the DISC level [26–28] and cleave Bid [29–32] allowing cytochrome c release from the mitochondria [29,30], which is a crucial event for FasL-induced caspase activation and apoptosis [25]. It has been recently reported that internalization of the Fas receptor is required for efficient DISC formation and apoptosis induction in type 1 cells but not in type 2 cells [33]. Fas stimulation has been also reported to activate a caspase-independent pathway involving the serine/threonine kinase RIP1 (Receptor Interacting Protein) as an effector molecule [34]. The signaling pathway activated by RIP is largely unknown and likely involves ROS production, and leads to a necrotic form of cell death rather than apoptosis [34].

A growing body of evidence supports the involvement of ceramide or its metabolites in stress-induced caspase-dependent and -independent cell death (for a review, see [35]). This ceramide-induced cell death is inhibited by over-expression of Bcl-2 or Bcl-xL, suggesting the involvement of mitochondrial events [35]. Moreover, we demonstrated that caspase-9-deficiency impairs ceramide-induced apoptosis in Jurkat T cells [36]. Ceramide has been recently proposed as a mediator in TNF-induced caspase-independent cell death in various cell lines [22]. In this context, ceramide production involved RIP1 [22].

Ceramide is synthesized within the endoplasmic reticulum and converted in the Golgi to sphingomyelin and glucosylceramide by SMS and GCS (GlucosylCeramide Synthase), respectively. Both enzymes are capable of negatively regulating intracellular ceramide concentrations and are inhibited upon various stress conditions, which trigger ceramide increase and cell death [35]. Accordingly, we have provided evidence that SMS1 and, albeit to a lesser extent, SMS2, behave as anti-apoptotic enzymes in Fas signaling, most likely through their ability to metabolize the pro-apoptotic ceramide into sphingomyelin [37]. However, anti-Fas induced apoptosis is partly impaired in a murine leukemia cell line deficient in SMS, and over-expression of SMS1 restores full caspase activation and cell death [38]. Thus, a sustained inhibition of SMS might alter membrane composition and properties through SM depletion and confer cell death resistance, whereas a transient inhibition of SMS could be involved in ceramide generation and apoptosis signaling [35,39].

Herein, we provide evidence that, in Jurkat cells and in PHA-activated T lymphocytes, D609 inhibits the activity of SMS and GCS, leading to a transient intracellular ceramide increase. Moreover, D609 enhances both caspase-dependent and -independent cell death in response to FasL and overcomes, to some extent, the resistance conferred by caspase inhibition and/or deficiency.

## 2. Materials and Methods

### 2.1. Reagents

Final concentrations or dilutions used of the following reagents are indicated: D609 (50 μg/mL or the indicated concentrations) was obtained from Sigma (Saint Quentin Fallavier, France); zVAD(OMe)-fmk (40 μM) was purchased from Bachem (Voisins-Le-Bretonneux, France); polyclonal anti-caspase-3 (10 μg/mL) was obtained from Dako (Trappes, France); monoclonal anti-PARP was purchased from Cell Signaling Technology (Saint Quentin en Yvelines, France) and used at 1/1000 dilution; monoclonal anti-β-actin (clone AC-15; 5 μg/mL) was obtained from Sigma; monoclonal anti-Bcl-xL (clone 2H12, 1 μg/mL) and anti-RIP (clone G322-2, 0.25 μg/mL) were purchased from BD Biosciences (Le Pont-De-Claix, France).

Human recombinant FasL was obtained from Abcys (Paris, France). Alternatively, mouse FasL produced in the supernatant of Neuro-2A cells stably transfected with a plasmid encoding FasL [40] was used. Similar data were obtained with mouse and human FasL.

### 2.2. Cell Lines

Parental Jurkat T leukemia cells (clone A3) and derived cell lines deficient in caspase-8 (clone I9-2) [41] were kindly provided by Dr. J. Blenis (Boston, MA). RIP1-deficient Jurkat cells and parental cells (derived from J77) [42] were kindly given by Dr. B. Seed (Boston, MA). Mock-transfected and Bcl-xL-over-expressing Jurkat cells (clone E6) [43] were obtained from Dr. C. Thompson (Chicago, IL). Human peripheral blood lymphocytes (PBL) were obtained from healthy donors after separation from heparinized venous blood by centrifugation (1500× g, 20 min) over Ficoll (Gibco, Cergy-Pontoise, France). Allowing cell adhesion to the flask for 4 h eliminated adherent cells. The remaining cells (*i.e.*, PBL) were cultured for 6 days with 1 μg/mL phytohemagglutinin (PHA) (Sigma) in the presence of 20 U/mL IL-2 (a kind gift from Sanofi Aventis, Toulouse, France). Cells were cultured in RPMI medium containing Glutamax and 10% heat-inactivated FCS.

### 2.3. Flow Cytometry Analyses

Phosphatidylserine (PS) externalization was evaluated by labeling cells with Annexin V-FITC (250 ng/mL) and propidium iodide (12.5 μg/mL) (Immunotech, Marseille, France) for 10 min at 4 °C. Hypodiploidy was detected by washing cells in PBS and permeabilization in ethanol (70%) for 10 min at −20 °C. Cells were next incubated for 30 min at 37 °C with RNase (1 μg/mL) and propidium iodide (0.1 mg/mL). The percentage of hypodiploid cells carrying a DNA content below that of cells in G0/G1 was quantified by flow cytometry. Analyses were performed on a FACScan (Becton Dickinson, Le Pont de Claix, France) cytometer [31].

### 2.4. Protein Extraction and Western Blotting Analyses

For total protein extraction, 5 × 10^6^ cells were lysed for 30 min on ice in a buffer containing 50 mM HEPES, pH 7.5, 150 mM NaCl, 10% glycerol, 1% Triton X-100, 0.5% deoxycholate, 1 mM NaVO_4_, 10 μM β-glycerophosphate, 50 mM NaF, 1 mM phenylmethylsulfonyl fluoride, 10 μg/mL leupeptin, 2 μg/mL pepstatin A and 10 μg/mL aprotinin. Samples were centrifuged at 10,000× g at 4 °C for 10 min. Supernatants were collected and protein content determined by the Bradford method (Biorad). For Western blot analyses, equal amounts of proteins were separated on 15% SDS-PAGE.

### 2.5. Determination of SMS and GCS Activities in Jurkat Cells

Jurkat cells were washed in PBS to remove serum and further incubated in RPMI medium containing 2.5 μM C_6_-NBD-ceramide (solubilized in ethanol) (Sigma). After incubation at 37 °C for two hours, cells were washed in PBS and centrifuged, and pellets were immediately frozen at −20 °C. Cell pellets were suspended in 0.2 mL of distilled water, and disrupted at 4 °C by brief sonication. After an aliquot had been taken for protein determination, lipids were extracted by adding 0.850 mL of chloroform/methanol (2:1, v/v). After centrifugation (1000× g, 10 min), the lower phases were dried under nitrogen and resolved by analytical thin layer chromatography (TLC) (Merck TLC Silica gel 60) developed in chloroform/methanol/30% ammonia (70:30:5, v/v/v). C_6_-NBD-ceramide, C_6_-NBD-GlcCer and C_6_-NBD-sphingomyelin were eluted from the silica and quantified spectrofluorometrically (L_ex_ = 470 nm and L_em_ = 560 nm) [44].

### 2.6. Ceramide Measurement

Lipids were extracted as described above. Ceramide mass was measured as previously reported [45] using *E. coli* membranes as a source of diacylglycerol kinase. Radioactive ceramide-1-phosphate was isolated by TLC (Whatman LK6D TLC plates) using chloroform/acetone/methanol/acetic acid/water (50:20:15:10:5, v/v/v/v/v).

### 2.7. Fluorogenic DEVD Cleavage Enzyme Assays

After incubation with FasL, cells were sedimented and caspase-like activities were assessed using Ac-DEVD-AMC (Bachem) as described elsewhere [46].

### 2.8. Morphological Analysis

Cells were co-incubated with propidium iodide (2 μg/mL) (Sigma, Lisle d’Abeau, France) and Syto 13 (2.5 μM) (Molecular Probes, Leiden, Netherlands) for 15 min at 37 °C and analyzed under a Leica fluorescence-equipped microscope [31,47]. At least 300 cells were examined.

### 2.9. Statistical Analysis

Results are expressed as means ± S.E.M., and are averages of at least three values per experiment. Mean values were compared using the Student’s *t*-test. Differences were considered statistically significant when *p* < 0.05 (as indicated by an asterisk on the figures; n.s.: not significant).

## 3. Results

### 3.1. Inhibition of SMS and GCS Activities by D609 Triggers Ceramide Increase and Cell Death in Jurkat Cells

**Figure 1:**
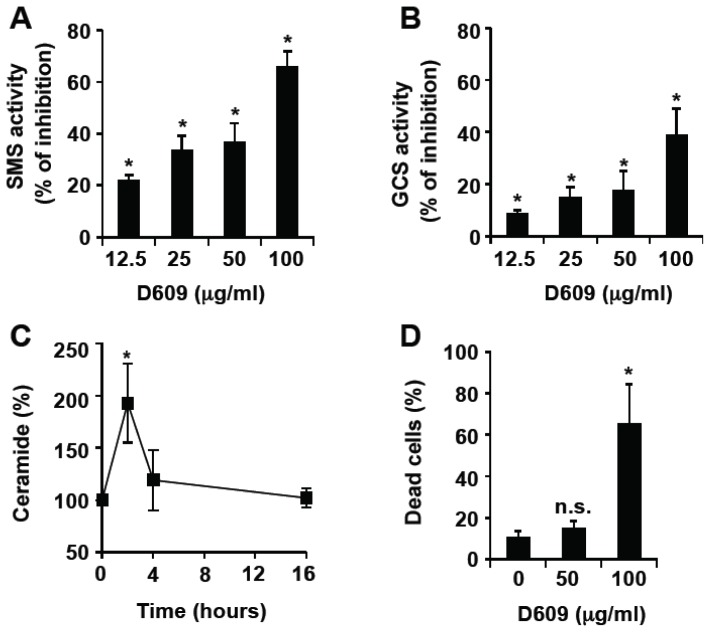

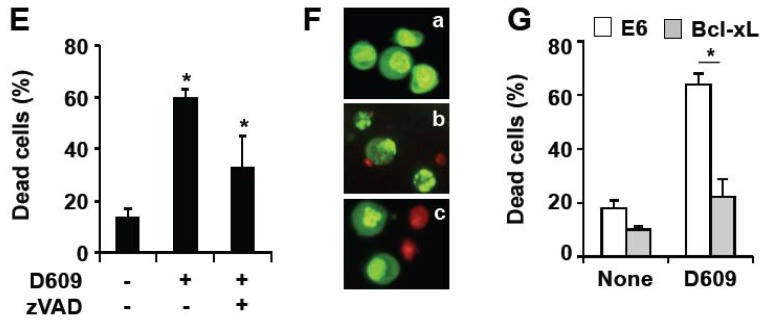
D609 inhibits SMS and GCS activities, and triggers ceramide increase and subsequent cell death in Jurkat cells. (**A**,**B**) Jurkat cells (clone A3) were incubated in the presence or absence of the indicated concentrations of D609 for 1 h and further incubated for 2 h in the presence of 2.5 μM C_6_-NBD-ceramide. SMS (**A**) and GCS (**B**) activities were determined by quantifying fluorescent SM and GlcCer. Results are expressed as the % of inhibition of the activities measured in the absence of D609. (**C**) A3 cells were incubated for the indicated times with 50 μg/mL D609 and ceramide content was quantified. (**D**–**G**) Cells were incubated for 16 h in the presence or absence of D609 (100 μg/mL or the indicated concentrations) and cell death was evaluated by microscopy analysis (**F**) or quantified by flow cytometry after annexin-V-FITC and propidium iodide labeling (**D**,**E**,**G**). At this incubation time, most of the annexin-V positive cells were also stained by propidium iodide and considered as dead cells. (**E**) Cells were pre-incubated for 1 h in the presence of 40 μM zVAD-fmk and further incubated for 16 h with 100 μg/mL D609 as indicated. (**F**) A3 cells were incubated with control medium (**a**), containing 100 μg/mL D609 (**b**) or a combination of 40 μM zVAD-fmk and 100 μg/mL D609 (**c**). After 16 h, cells were stained with Syto-13 (green probe) and propidium iodide and analyzed under a fluorescent microscope. **G**, Mock-transfected E6 Jurkat cells (E6) and Bcl-xL-over-expressing E6 cells (Bcl-xL) were incubated in the presence (D609) or absence (None) of 100 μg/mL D609 for 16 h. (**A**–**E**,**G**) Values are means ± S.E.M. of three independent experiments. (**F**) Pictures are representative of three independent experiments.

D609 has been previously shown to inhibit SMS activity in SV40-transformed fibroblasts and leukemia cells [15–18]. We first monitored D609 effect on SMS and GCS activities in Jurkat cells by measuring the conversion of a fluorescent ceramide analog to SM and GlcCer. D609 not only inhibited SMS (Figure 1A) but also, albeit to a lesser extent, GCS (Figure 1B) in a dose-dependent manner. Moreover, treatment with 50 μg/mL (*i.e.*, 187.5 μM) D609 resulted in a transient elevation of endogenous ceramide levels up to 200% of the basal value at 2 h, followed by a decline to basal level by 4 h (Figure 1C) with no significant toxicity induction (Figure 1D). 100 μg/mL (*i.e.*, 375 μM) D609 triggered cell death (Figure 1D) that was not totally inhibited by the broad-spectrum caspase inhibitor zVAD-fmk (Figure 1E), under conditions that abolished caspase-3 and PARP cleavage (data not shown). This indicates that D609 can induce both caspase-dependent and -independent Jurkat cell death. Whereas D609 alone triggered apoptosis, as evidenced by nuclear fragmentation, D609-induced cell death in the presence of zVAD-fmk was associated with marginal chromatin condensation and necrotic features (Figure 1F). Moreover, cell death was strongly impaired by Bcl-xL over-expression, suggesting that mitochondrial events are involved in D609-induced toxicity (Figure 1G).

### 3.2. D609 Stimulates FasL-Induced Caspase Activation and Apoptosis in Jurkat Cells

**Figure 2:**
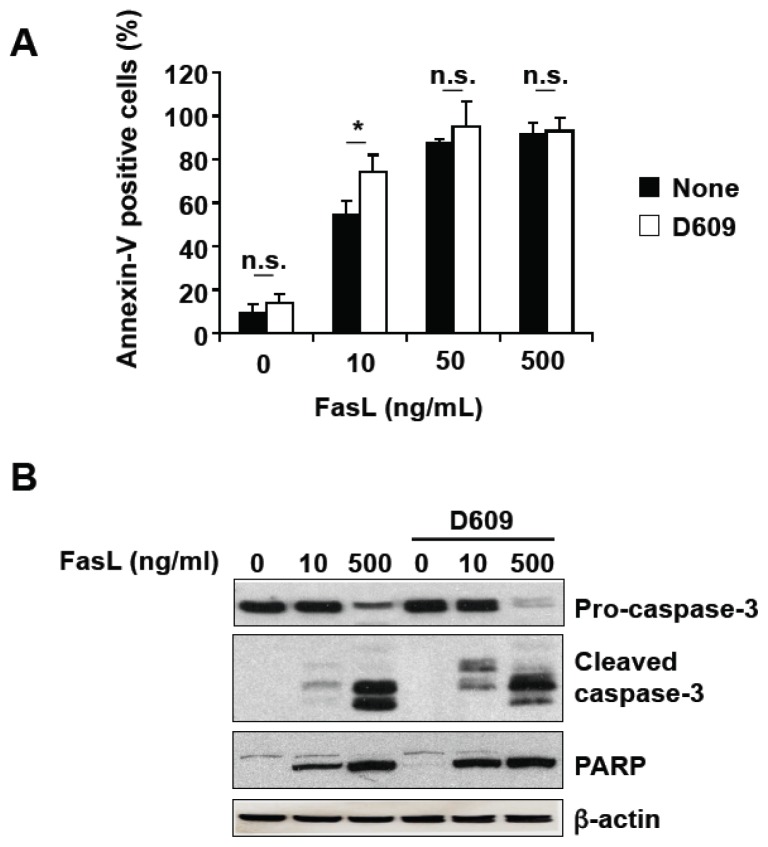
D609 enhances FasL-induced caspase activation and cell death in Jurkat cells. Jurkat cells (clone A3) were pre-incubated in the presence (white bars) or absence (black bars) of 50 μg/mL D609 for 1 h and further incubated for 16 h in the presence of the indicated FasL concentrations. (**A**) Cell death was next evaluated by flow cytometry after annexin-V-FITC and propidium iodide labeling. Most of the annexin-V positive cells were not stained by propidium iodide even in the presence of D609. Values are means ± S.E.M. of three independent experiments. (**B**) Caspase activation was assessed by Western blot using anti-caspase-3 or anti-PARP antibodies. Anti-β-actin antibody was used as a control. Data are representative of two independent experiments.

Controversy exists as to the capacity of D609 to modulate Fas-cross linking-induced cell death [16,23,24]. We re-evaluated D609 effect in Fas cell death signaling in response to FasL (Figure 2A). At a sub-toxic concentration, D609 significantly enhanced Fas-engagement-induced PS externalization (Figure 2A). In response to FasL, caspase activation was increased by D609 as evaluated by Western blot using anti-caspase-3 and anti-PARP antibodies (Figure 2B).

### 3.3. D609 Enhances FasL-Induced Caspase-Independent Cell Death and Overcomes Caspase-8 and RIP Deficiency

**Figure 3:**
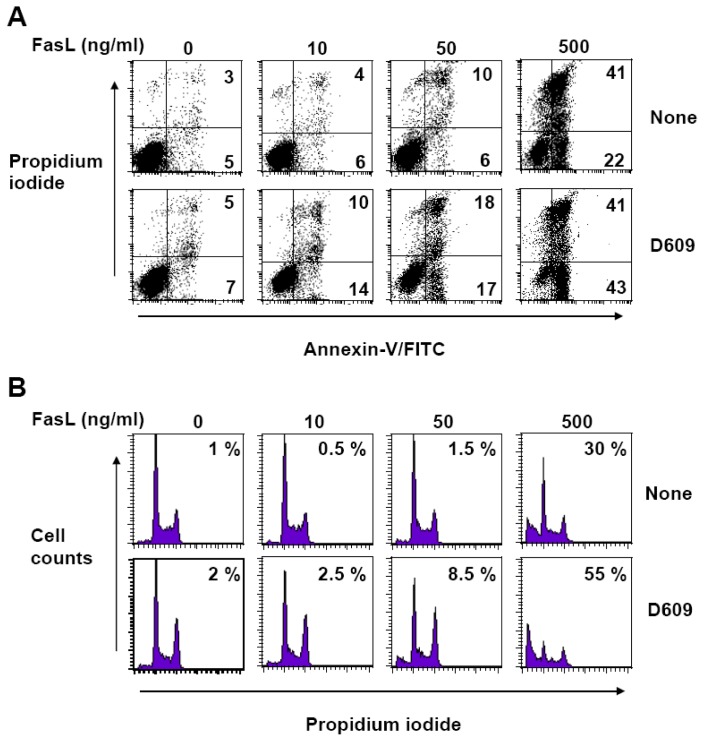
D609 enhances FasL-induced cell death in caspase-8-deficient Jurkat cells. Caspase-8-deficient Jurkat cells (clone I9–2) were pre-incubated in the presence (D609) or absence (None) of 50 μg/mL D609 for 1 h and further incubated for 16 h in the presence of the indicated FasL concentrations. (**A**) Cell death was next evaluated by flow cytometry after annexin-V-FITC and propidium iodide labeling. The percentage of annexin-V-FITC-positive and propidium iodide-negative cells is indicated in the lower right quadrants. The percentage of propidium iodide positive cells is indicated in the upper right quadrants. (**B**) The percentages of hypodiploid cells were determined by flow cytometry. (**A**,**B**) Data are representative of two independent experiments.

In Jurkat cells, caspase-8 plays a pivotal role in initiating caspase cascade activation in response to Fas engagement [41]. As compared to wild-type Jurkat cells (see Figure 2), toxicity was strongly impaired in caspase-8-null Jurkat cells in response to 10–50 ng/mL FasL whereas 500 ng/mL FasL triggered substantial cell death as evaluated by annexin-V and propidium labeling (Figure 3A) and DNA content analysis (Figure 3B). D609 sensitized caspase-8-deficient Jurkat cells to all doses of FasL, indicating that D609 acts downstream of caspase-8 and overcomes, to some extent, caspase-8 deficiency (Figure 3).

**Figure 4:**
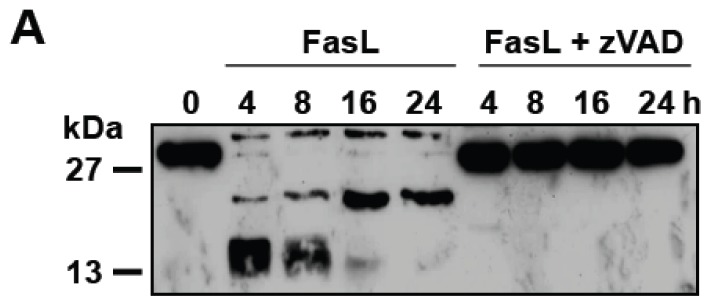

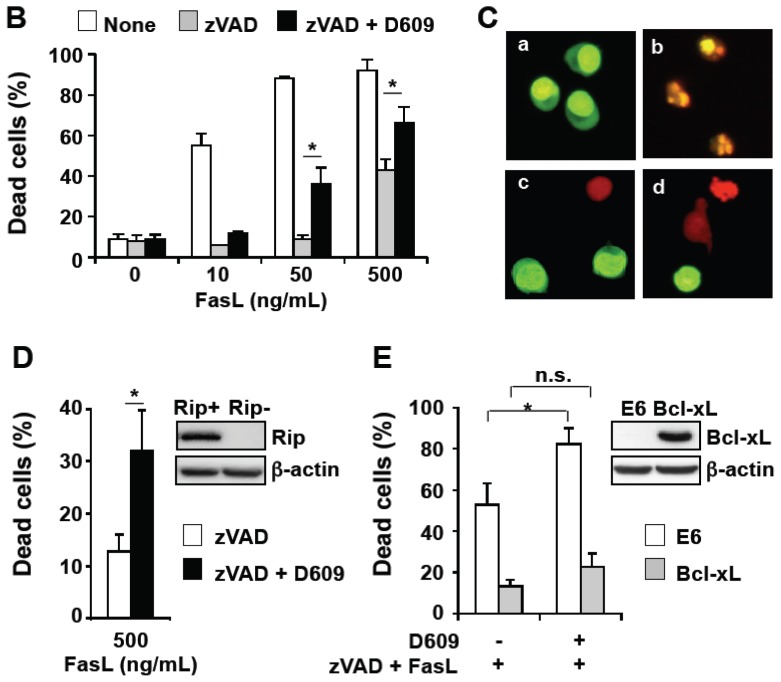
D609 overcomes zVAD-fmk-induced resistance of Jurkat cells to FasL. Jurkat cells were pre-incubated for 1 h with or without 40 μM zVAD-fmk or a combination of 40 μM zVAD-fmk and 50 μg/mL D609. Cells were further incubated for 16 h (or the indicated times) with or without FasL (500 ng/mL or the indicated concentrations). Experiments were carried out using Jurkat cells (clone A3) (**A**–**C**), J77 parental (RIP+) and RIP-deficient (RIP−) Jurkat cells (**D**) and mock-transfected E6 Jurkat cells (E6) and Bcl-xL-over-expressing E6 cells (Bcl-xL) (**E**). (**A**) Caspase-3 activation was evaluated by Western blot. Data are representative of two independent experiments. (**B**,**D**,**E**) Cell death was evaluated by flow cytometry after annexin-V-FITC and propidium iodide labeling. Under these experimental conditions, most of the dead cells were labeled by both annexin-V-FITC and propidium iodide. (**C**) Cells were incubated with control medium (**a**), or medium containing FasL (**b**), zVAD-fmk plus FasL (**c**) or a combination of zVAD-fmk, D609 and FasL (**d**). After 16 h, cells were stained with Syto-13 (green probe) and propidium iodide and analyzed under a fluorescent microscope. (**D**) Western blot analyses were performed using anti-RIP and anti-β-actin antibodies. (**E**) Western blot analyses were assessed using anti-Bcl-xL and anti-β-actin antibodies. (**B**,**D**,**E**) Values are means ± S.E.M. of three independent experiments. (**C**) Pictures are representative of two independent experiments.

We next evaluated the effect of D609 in FasL-induced cell death in the presence of zVAD-fmk. In response to 500 ng/mL FasL, zVAD-fmk totally prevented caspase-3 cleavage (Figure 4A) and the increase in caspase activity towards Ac-DEVD-AMC and Ac-IETD-AMC [48], which are substrates for effector and initiator caspases, respectively. FasL-induced cell death was completely abolished by zVAD-fmk when Jurkat cells were incubated with low doses of FasL (*i.e.*, 10 and 50 ng/mL) (Figure 4B). However, in agreement with previous reports [31,34,49], toxicity was only partly inhibited by zVAD-fmk in response to a higher FasL concentration (*i.e.*, 500 ng/mL), indicating that Fas engagement activates both caspase-dependent and -independent cell death pathways (Figure 4B). The addition of D609 not only significantly sensitized Jurkat cell death induced by a high FasL concentration (500 ng/mL), but also by-passed zVAD-fmk-mediated resistance toward FasL concentration as low as 50 ng/mL (Figure 4B). Whereas FasL alone triggered apoptosis as evidenced by nuclear fragmentation, FasL-induced cell death was associated with some necrotic features (*i.e.*, marginal chromatin condensation and membrane permeability increase) when cells were incubated with zVAD-fmk in the presence or absence of D609 (Figure 4C). Given the critical role of RIP in FasL-induced caspase-independent cell death [34], the effect of D609 was tested in RIP-deficient Jurkat cells (Figure 4D). D609 overcame RIP deficiency, restoring the capacity of FasL to promote cell death in RIP-null Jurkat cells in the presence of zVAD-fmk (Figure 4D). The sensitizing effect of D609 in FasL-induced caspase-independent cell death was strongly impaired by Bcl-xL over-expression, suggesting the involvement of mitochondrial events (Figure 4E).

### 3.4. D609 Increases FasL-Induced Caspase-Independent Ceramide Production

**Figure 5:**
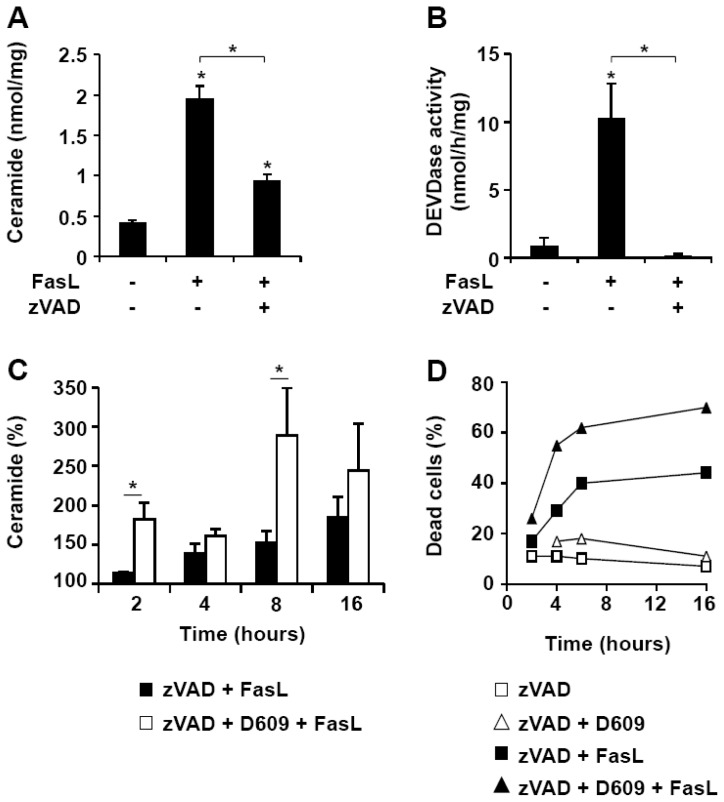
D609 enhances FasL-induced caspase-independent ceramide increase and cell death. Jurkat cells (clone A3) were pre-incubated in the presence or absence of 40 μM zVAD-fmk for 1 h and further incubated with or without FasL (500 ng/mL) for 16 h or the indicated times. Ceramide level (expressed as nmol of ceramide per mg of protein) (**A**) and caspase activity toward Ac-DEVD-AMC (**B**) were measured. (**C**) Cells were pre-incubated for 1 h with 40 μM zVAD-fmk (black bars) or a combination of 40 μM zVAD-fmk and 50 μg/mL D609 (white bars). Cells were further incubated with 500 ng/mL FasL for the indicated times and ceramide concentration was measured. Data are expressed as the percentage of values measured in cells incubated with zVAD-fmk alone. (**A**–**C**) Values are means ± S.E.M. of three independent experiments. (**D**) Cells were pre-incubated for 1 h with 40 μM zVAD-fmk in the presence (triangles) or absence (squares) of 50 μg/mL D609. Cells were further incubated for the indicated times with (solid symbols) or without (empty symbols) 500 ng/mL FasL. Cell death was evaluated by flow cytometry after annexin-V-FITC and propidium iodide labeling. Under these conditions, most of the dead cells were labeled by both annexin-V-FITC and propidium iodide. Data are representative of two independent experiments.

In agreement with previous reports showing that Fas cross-linking triggers ceramide generation in Jurkat cells [16,46,50,51], 500 ng/mL FasL induced a four-fold increase in intracellular ceramide levels (Figure 5A). This ceramide elevation was only partly affected by zVAD-fmk (Figure 5A) whereas caspase activation, as evaluated by the measurement of DEVDase activity, was suppressed (Figure 5B). Ceramide increased in a time-dependent manner and was concomitant with cell death (Figure 5C,D). D609 enhanced both ceramide generation (Figure 5C) and cell death (Figure 5D) at all times. Altogether, our data indicate that D609 stimulates FasL-induced caspase-independent ceramide increase and toxicity in Jurkat cells.

### 3.5. D609 Enhances FasL-Induced Cell Death in T Lymphocytes

**Figure 6:**
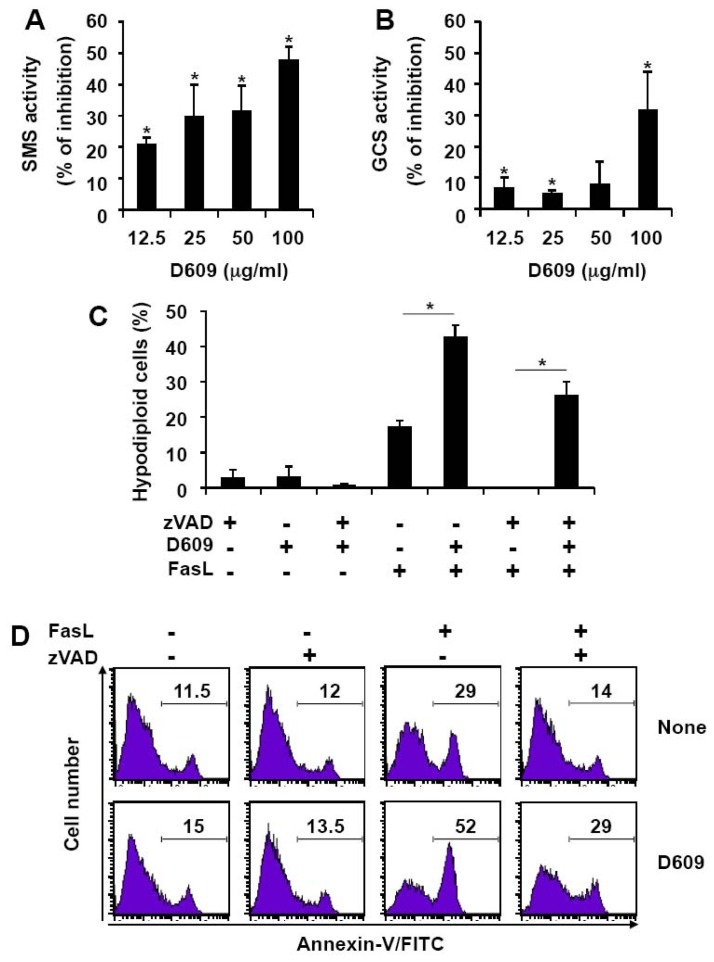
D609 inhibits SMS and GCS activities and enhances FasL-induced cell death in PHA-activated human T lymphocytes. (**A**,**B**) Human PBL, derived from three healthy volunteers, were cultured for 6 days in the presence of PHA. Cells were next pre-incubated in the presence or absence of the indicated concentrations of D609 for one hour and further incubated for 2 h in the presence of 2.5 μM C6-NBD-ceramide. SMS (**A**) and GCS (**B**) activities were determined. (**C**,**D**) PHA-activated PBL were pre-incubated for 1 h with or without 40 μM zVAD-fmk and 50 μg/mL D609 as indicated. Cells were further incubated for 16 h in the presence or absence of 500 ng/mL FasL. (**C**) The percentages of hypodiploid cells were determined by flow cytometry. Basal hypodiploidy in untreated cells did not exceed 15% in all conditions and was subtracted from the values. Values are means ± S.E.M. of three independent experiments. (**D**) PS externalization was measured by flow cytometry after annexin-V/FITC and propidium iodide staining. Analysis was restricted to propidium iodide negative cells to exclude cellular debris derived from non-activated lymphocytes. Numbers indicate the percentage of cells labeled with annexin-V/FITC. Data are representative of three independent experiments.

We next evaluated the effect of D609 in primary T cells. Similarly to Jurkat cells, D609 inhibited SMS and, to a lesser extent, GCS in PHA-activated T lymphocytes in a dose-dependent manner (Figure 6A,B). FasL-induced cell death, as evaluated by the increase of hypodiploid cells (Figure 6C) and PS externalization (Figure 6D), was significantly stimulated by D609. Whereas zVAD-fmk completely abrogated cell death increase in response to FasL, D609 restored FasL-induced cell death and, thus, overcame caspase inhibition-induced resistance of T lymphocytes.

## 4. Discussion

The present study demonstrates that D609 acts as a potent inhibitor of SMS and, to a lesser extent, of GCS, in T cells. At a non-toxic concentration, D609 dose enhances FasL-induced caspase-dependent and -independent cell death. Moreover, D609 somehow overcomes caspase-8 and RIP deficiency-induced resistance to FasL.

D609 has often been used as a specific inhibitor of PC-PLC [2,4,10–13]. As described by others [15,16,18], we show that D609 is also capable of inhibiting SMS activity (Figures 1A and 6A). In addition, GCS activity decreases in the presence of D609 (Figures 1B and 6B). Thus, D609 cannot be considered as a specific inhibitor of PC-PLC and SMS and is likely able to inhibit other lipid metabolizing enzymes, such as GCS or phospholipases, including PC-PLD and PE-PLC [52]. Thus, D609 probably acts on multiple metabolic and signaling pathways, and the molecular mechanisms involved are not fully established.

In our experimental setting, a non-toxic D609 concentration triggered a transient increase of intracellular ceramide, most likely as a consequence of the inhibition of ceramide conversion into its immediate metabolites, *i.e.*, SM and GlcCer. Ceramide levels returned to basal values at 4 h, possibly due to the ceramide metabolism into complex sphingolipids and/or ceramide catabolism into sphingosine. We have recently documented that ceramide activates the mitochondrial pathway leading to cytochrome c release and caspase-9 activation in Jurkat cells [36]. As a matter of fact, Bcl-xL over-expression inhibited D609 effects, strongly indicating the involvement of mitochondria. Thus, ceramide appears to be a good candidate for mediating D609 effects, although a previous study reported that D609-induced cell death is not affected when ceramide increase is pharmacologically inhibited by the use of fumonisin B1 or L-cycloserine, both known to block *de novo* ceramide synthesis in the endoplasmic reticulum [53]. However, it is conceivable that, despite reducing the total intracellular level of ceramide with fumonisin B1 or L-cycloserine, D609 exerts its effects by increasing a specific pool of ceramide, different from that of *de novo* synthesis. Thus, although we cannot establish a definitive link between D609-induced ceramide production and cell death, we cannot rule out the possibility that ceramide elevation in the Golgi, the plasma membrane and/or the nucleus as a consequence of inhibition of SMS and GCS, is involved in D609 cytotoxic effects. Subcellular localization of ceramide production determines the capacity of ceramide to act as a biological molecule in cell death [35]. For instance, we previously reported that lysosomal ceramide is not involved in Fas-cross-linking and stress-induced cell death [54]. Ectopic-expression of a bacterial SMase induces cell death only when it is targeted to the mitochondria [55].

To determine whether GCS inhibition is an important event for D609 effects, we used a more specific GCS inhibitor, *i.e.*, PDMP, which has been shown to enhance anti-cancer drug-induced apoptosis in some cancer cells [56]. Pre-treatment of Jurkat cells (from 1 h to 16 h) with 10 μM PDMP resulted in a potent (more than 90%) inhibition of GCS activity, but had no effect on FasL-induced cell death in the presence or absence of zVAD-fmk (data not shown). This observation suggests that the inhibition of GCS activity by D609 is unlikely to be sufficient for sensitizing cells to FasL. Of note, Tepper and co-workers previously reported that GCS does not modulate ceramide generation and Jurkat cell death in response to Fas stimulation [57]. However, the possibility that GCS inhibition contributes to D609 effects in FasL-induced ceramide generation and cell death, together with SMS inhibition and possibly other metabolic alterations, cannot be excluded.

Two different genes encoding SMS have been cloned so far. The corresponding proteins, SMS1 and SMS2, are mainly localized at the Golgi and at the plasma membrane, respectively [17]. Both enzymes can be inhibited by D609, the extent of inhibition for SMS1 being greater than for SMS2 [17]. Moreover, Okazaki’s group has recently reported that Fas cross-linking leads to the inhibition of a nuclear SMS, leading to ceramide increase into the nucleus [16]. Furthermore, D609 is also able to inhibit SMS into the nucleus and to enhance Fas-induced nuclear ceramide accumulation and apoptosis [16]. The inhibition of nuclear SMS has been proposed as a consequence of caspase activation in response to Fas stimulation [16]. More recently, we have shown that SMS1 knockdown sensitizes Jurkat cells (which express SMS1 but not SMS2) to FasL-induced apoptosis [37], further indicating that SMS behave as anti-apoptotic enzymes in Fas signaling. The present study demonstrates that D609 enhances FasL-induced caspase activation and apoptosis. Thus, it is likely that caspase-dependent ceramide increase, possibly within the nucleus and/or the Golgi, acts as a positive amplification loop in caspase cascade activation and apoptosis induction. Moreover, the sensitization effect of D609 was observed both in Jurkat and activated T cells, which are respectively type 2 and type 1 cells as regards Fas signaling pathways. Thus, our data suggest that different cell types could be sensitized to FasL by D609.

In our hands, D609 also promotes FasL-induced ceramide accumulation and cell death in the presence of zVAD-fmk, a widely-used broad-spectrum caspase inhibitor (see Figures 4C and 5C). D609 also enhanced FasL-induced cell death in caspase-8-deficient Jurkat cells (see Figure 3). Thus, our results indicate that D609 effects in Fas signaling are not restricted to the caspase-dependent pathway. In the absence of D609, zVAD-fmk resistant (*i.e.*, caspase-independent) cell death occurs only in the presence of high FasL concentration and requires the RIP kinase [34]. D609 not only sensitizes Jurkat cells to caspase-independent cell death in response to low FasL concentration (*i.e.*, 50 ng/mL), but also by-passes RIP deficiency, restoring the capacity of FasL to kill RIP-null Jurkat cells in the presence of zVAD-fmk. Similarly, D609 overcomes zVAD-fmk-mediated resistance of PHA-activated T lymphocytes to FasL. In humans, autoimmune lymphoproliferative syndromes (ALPS) develop as a consequence of alteration(s) in the Fas/FasL system. Some defects in the caspase-dependent pathway, as the result of gene mutations affecting the catalytically active sites of either caspase-8 or -10, can be responsible for ALPS [58]. A major finding presented here is the ability of D609 to enhance FasL-induced cell death or to restore it in cells where the death cascade is impaired. Thus, the use of D609 or derivatives [59] may represent a promising strategy, at least in some cases, for the treatment of patients affected with ALPS.
